# Microglial Activation and Oxidative Stress in PM_2.5_-Induced Neurodegenerative Disorders

**DOI:** 10.3390/antiox11081482

**Published:** 2022-07-29

**Authors:** Jie Song, Keyang Han, Ya Wang, Rongrong Qu, Yuan Liu, Shaolan Wang, Yinbiao Wang, Zhen An, Juan Li, Hui Wu, Weidong Wu

**Affiliations:** 1 School of Public Health, Xinxiang Medical University, Xinxiang 453003, China; 171058@xxmu.edu.cn (J.S.); 50200209002@stu.xxmu.edu.cn (K.H.); 50200102025@stu.xxmu.edu.cn (R.Q.); 50200209006@stu.xxmu.edu.cn (Y.L.); 50200209008@stu.xxmu.edu.cn (S.W.); 151049@xxmu.edu.cn (Y.W.); 151037@xxmu.edu.cn (Z.A.); 171019@xxmu.edu.cn (J.L.); wuhui@xxmu.edu.cn (H.W.); 2 Nursing School, Zhenjiang College, Zhenjiang 212028, China; wangya2021@zjc.edu.cn

**Keywords:** PM_2.5_, microglia, oxidative stress, neuroinflammation, neurodegeneration

## Abstract

Fine particulate matter (PM_2.5_) pollution remains a prominent environmental problem worldwide, posing great threats to human health. The adverse effects of PM_2.5_ on the respiratory and cardiovascular systems have been extensively studied, while its detrimental effects on the central nervous system (CNS), specifically neurodegenerative disorders, are less investigated. Neurodegenerative disorders are characterized by reduced neurogenesis, activated microglia, and neuroinflammation. A variety of studies involving postmortem examinations, epidemiological investigations, animal experiments, and in vitro cell models have shown that PM_2.5_ exposure results in neuroinflammation, oxidative stress, mitochondrial dysfunction, neuronal apoptosis, and ultimately neurodegenerative disorders, which are strongly associated with the activation of microglia. Microglia are the major innate immune cells of the brain, surveilling and maintaining the homeostasis of CNS. Upon activation by environmental and endogenous insults, such as PM exposure, microglia can enter an overactivated state that is featured by amoeboid morphology, the over-production of reactive oxygen species, and pro-inflammatory mediators. This review summarizes the evidence of microglial activation and oxidative stress and neurodegenerative disorders following PM_2.5_ exposure. Moreover, the possible mechanisms underlying PM_2.5_-induced microglial activation and neurodegenerative disorders are discussed. This knowledge provides certain clues for the development of therapies that may slow or halt the progression of neurodegenerative disorders induced by ambient PM.

## 1. Introduction

Air pollution is comprised of particulate matter (PM), gases, organic compounds, and metals derived from both human activity and natural sources. PM is the most widespread health threat and has been strongly implicated in diverse diseases [1]. An important contributor to PM is traffic-related air pollution (TRAP), mostly ascribed to diesel exhaust particles (DEP) [2]. Ambient PM can be split up in several size fractions based on the aerodynamic diameter: coarse PM (2.5–10 μm), fine PM (<2.5 μm, PM_2.5_), and ultrafine PM (<0.1 μm, UFPM) [3]. PM_2.5_ exhibits tempo-spatial variations of complex components, such as bacteria lipopolysaccharides (LPS), carbon-containing particles, sulfate, nitrate, ammonium salt, and heavy metals [4,5]. PM_2.5_ and UFPM are of particular concern, as these particles can enter systemic circulation and be distributed in the brain and other organs, posing significant potential danger to human health [6]. Globally, there are approximately 6.5 million excess deaths attributable to ambient PM_2.5_ pollution annually [7]. Recent evidence indicates that PM_2.5_ air pollution, in addition to causing respiratory and cardiovascular diseases, also negatively affects the brain and contributes to central nervous system (CNS) diseases [1].

Since anatomopathological evidence from canine and human residents in Mexico City was reported in the early 2000s [8,9,10], the neurotoxicity of PM_2.5_ has received much attention. Many studies have reported association of PM_2.5_ exposure with neurological disorders, such as stroke, dementia, Alzheimer’s disease (AD), Parkinson’s disease (PD), and mild cognitive impairment [11]. Thus far, the mechanisms underlying PM_2.5_-induced neurological disorders have not been well elucidated, with oxidative stress and neuroinflammation being two major recognized ones [12]. Due to its high metabolic demands, high energy use, high lipid content, widespread axonal and dendritic networks, and low levels of endogenous antioxidants, the brain is more susceptible to oxidative stress [13], the latter refers to the imbalance between the production of free radicals, such as reactive oxygen species (ROS) and reactive nitrogen species (RNS), and the antioxidant defense systems, which can damage cellular biomolecules, including lipids, proteins, and DNA [14]. Oxidative stress has been proposed as a hallmarker and major driving force for neurodegeneration [15]. 

Microglia are the principal players in the brain’s innate immune response [16]. Emerging evidence from recent studies has suggested that microglial activation, oxidative stress, neuroinflammation, cerebrovascular damage, and abnormal protein aggregates may play critical roles in the pathogenesis of neurodegenerative disorders triggered by ambient PM_2.5_ [1,17,18]. There are a few reviews elaborating on the association of PM exposure with adverse neurological effects, especially neuroinflammation [12,17,18,19,20], but with none centering on microglial involvement. This review systemically summarizes the evidence of PM_2.5_-induced oxidative stress and neurodegenerative disorders from postmortem examinations, epidemiological investigations, animal experiments, and in vitro studies, with an emphasis on microglial implications in these pathophysiological events. In addition, the potential mechanisms underlying PM_2.5_-induced microglial activation and associated oxidative stress and neurodegenerative disorders are also discussed. 

## 2. Microglia: Physiological and Pathological Characteristics

Glial cells account for more than 90% of cells in the human brain and are divided into two populations: the macroglia (i.e., astrocytes and oligodendrocytes) and microglia [21]. Microglia originate from immature yolk sac progenitor cells and are present in significant numbers in normal brains, but their density varies by brain region in the adult human and mice [22,23,24]. More microglia are found in the cortex than in the white matter, with the highest concentrations found in the hippocampus, olfactory telencephalon, basal ganglia, and substantia nigra [25,26]. Such distribution may explain the vulnerability of these brain areas. 

**Figure 1:**
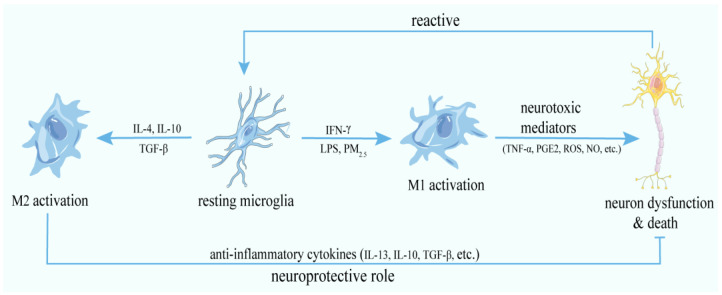
M1/M2 phenotypes and functions of microglia. The resting microglia can be activated by toxic mediators, such as IFN-γ, LPS, and PM_2.5_, and display a M1 phenotype. In this condition, microglia induce neurotoxicity via release of neurotoxic mediators (TNF-α, PGE2, ROS, NO, etc.). The factors secreted by the dead or damaged neurons in turn exacerbate the chronic activation of microglia. Besides the M1 phenotype, in combination with IL-4, IL-10, and TGF-β, microglia could be induced into the M2 phenotype, which plays a neuroprotective role through the generation and release of anti-inflammatory cytokines (IL-13, IL-10, TGF-β, etc.).

Under resting conditions, microglia survey the microenvironment in real-time with their ramified, motile, fine, and long cellular processes [26]. Meanwhile, diverse neurotrophic factors are released from microglia and help to maintain neuronal cell survival and circuit formation [27]. In contrast to neurons, microglial cells have the ability to completely restore their population in the adult brain [28]. Microglia can be activated by endogenous disease proteins, cytokines, neuronal death, and environmental toxicants including components of air pollution [1]. Activated microglia in the CNS are heterogeneous and can be categorized into two opposite phenotypes: classical (M1) or alternative (M2) (Figure 1) [29]. The M1 phenotype characterized by amoeboid shape, high mobility, and strong phagocytic capacity is mainly induced by LPS, interferon-γ (IFN-γ), amyloid β (Aβ), and α-synuclein [30,31], and associated with the release of pro-inflammatory cytokines and chemokines, such as tumor necrosis factor-α (TNF-α), interleukin (IL)-6, IL-1β, IL-12, CC chemokine ligand-2 (CCL-2), monocyte chemoattractant protein-1 (MCP-1), and prosglandins [31,32,33], whose receptors are found on neurons, thus rendering neurotoxicity [34,35]. Moreover, activated microglia also over-express nicotinamide adenine dinucleotide phosphate (NADPH) oxidase (NOX) and inducible nitric oxide synthase (iNOS) that catalyze the generation of ROS and nitric oxide (NO), respectively [36], and a major histocompatibility complex-II that presents antigens, triggers and spreads further inflammatory response in surrounding microglial cells [28], integrins, co-stimulatory molecules, Fc receptors, and intracellular proteins (e.g., ionized calcium binding adapter molecule-1, Iba-1), contributing to neurological damage [37]. In contrast, the M2 phenotype characterized by thin cell bodies and branched processes can be induced by IL-4, IL-13, IL-10, or activated peroxisome proliferator-activated receptors γ (PPARγ) [38], resulting in the release of anti-inflammatory cytokines, such as IL-10, transforming growth factor-β (TGF-β), growth factors, colony stimulating factor−1 (CSF-1), neurotrophic growth factors, such as brain derived neurotrophic factor (BDNF), a neuroprotective status [38,39,40]. 

From the above, activated microglia show a broad spectrum of phenotypes ranging from the pro-inflammatory, potentially cytotoxic M1 to the anti-inflammatory, scavenging, and regenerative M2.

## 3. Evidence from Postmortem Examinations

The first histopathological evidence for a link between air pollution and neuropathology came from a necropsy study with canine residents naturally exposed to a highly polluted environment in Mexico City [41]. In this study, the increased expression of neuroinflammatory and oxidative stress biomarkers, including nuclear factor-κB (NF-κB) and iNOS in cortical endothelial cells occurred at ages 2 and 4 weeks of dogs with subsequent neurodegenerative changes, such as the impairment of the blood–brain barrier (BBB) and the extracellular deposition of Aβ peptide fibrils and intracellular neurofibrillary tangles (NFTs) in the olfactory epithelium (OE) and the olfactory bulbs (OB), as well as in subcortical and cortical structures [41]. In addition, dogs aged 8 months demonstrated pronounced inflammatory effects, diffuse Aβ plaques, and a significant increase in DNA damage in OB, frontal cortex, and hippocampus with ameboid microglia in the cortex and white matter [8,41]. Moreover, feral dogs inhabiting in Mexico City presented brain tissue damage and accumulated metals (nickel and vanadium) in a gradient fashion from OE > OB > frontal cortex, indicating the nose as portal of entry [41]. Notably, those alterations of the olfactory pathway were similar to the early olfactory pathology observed in AD.

The similar findings were also observed in autopsy examinations of human samples [9]. Adult human residents living in Mexico City presented an increased expression of inflammatory mediator cyclooxygenase-2 (COX-2) and the greater accumulation of neuronal and astrocytic Aβ_42_, the 42-amino acid form of Aβ, in frontal cortex, hippocampus, and OB [9]. In contrast, children and young adults exhibited a wide spectrum of neurodegenerative disorders, such as increases in microglial activation (CD68 and CD163), elevated pro-inflammatory proteins (COX-2 and IL-1β) and the innate immunity receptor CD14, accumulated AD or PD proteins (Aβ_42_ and α-synuclein), oxidative stress in frontal and infratentorial neurons and microglia (8-hydroxydeoxyguanosine (8-OHdG) and nitrotyrosine), and frontal BBB impairment, as well as the reduction of the neuroprotective cellular prion protein (PrPC) in the frontal cortex [42]. Intriguingly, metals (manganese, nickel, and chromium) were enriched in the frontal cortex with the high expression of COX-2, TGF-β, CD14, and IL-1β [42]. Additionally, those children in Mexico City had brainstem abnormalities, such as inflammation, α-synuclein and/or Aβ_42_, deposition, activated microglia, and reactive glial fibrillary acidic protein (GFAP) positive astrocytes throughout the brainstem [43]. Early olfactory abnormalities similar to the olfactory pathology in AD were also observed in Mexico City children [44]. It is noteworthy that Apolipoprotein E4 (APOE4) is the strongest genetic risk factor for AD [45], APOE4 carriers exposed to air pollution had greater hyperphosphorylated tau, diffused Aβ plaques, and more pronounced olfactory deficits than APOE3 carriers [42,46,47]. 

Taken together, the preliminary evidence of postmortem studies indicates that exposure to ambient air pollutants is associated with microglia activation, oxidative stress, and neurodegenerative alterations in brain tissues. However, due to the complex mixture of air pollution, the causative association of air pollution exposure with the observed neurological effects of CNS remains to be specified.

## 4. Evidence from Epidemiological Investigations

There is a growing body of epidemiological studies reporting ambient air pollution-associated neurodegenerative disorders (see Table 1 for details), such as cognitive decline, AD, and PD. Regarding cognitive alteration, an early important aspect of AD, many population-based studies with the elderly [48,49,50] have reported that PM air pollution, particularly TRAP, is consistently associated with declined cognitive abilities [51]. However, controlled animal studies in this aspect are still limited for further verification. 

Several other epidemiological studies found that exposure to PM_2.5_ was associated with a significant risk for AD [52,53,54,55,56], which was consistent with the pathological findings from the autopsy samples of individuals with AD-like pathologies in the highly polluted Mexico City. These observations were supported by a follow-up study displaying a decrease in Aβ_42_ levels in the cerebrospinal fluids (CSF) of Mexico City children [57], a very early change in AD [58]. In addition, Calderon-Garciduenas et al. recently reported elevated levels of non-phosphorylated tau in the CSF, a marker of AD axonal pathology, or increases in hyperphosphorylated tau and amyloid plaques in the OB of children and young adults in Mexico City [51,59]. Given that aging is a risk factor for neurodegenerative diseases [60], the aging brain is assumed to be particularly vulnerable to air pollution-induced neurotoxicity [61]. Therefore, it is not surprising to see an accelerated decline in episodic memory among older females with late-life exposure to PM_2.5_ [62].

A few studies have examined the association of ambient PM_2.5_ exposure with the risk for PD. However, the epidemiological results appear inconsistent. For example, one study showed opposite associations between ambient PM_2.5_ exposure and the incidence of PD among two populations from two locations with different severity of air pollution [63]. Another study found an association between the concentrations of ambient PM_2.5_ and PD risk in female never-smokers [64]. However, in a large prospective study of women, Palacios et al. did not see significant associations between PM_10_ or PM_2.5_ exposure and the incidence of PD [65].

Thus far, the epidemiological evidence specifically linking microglial activation to air pollution-induced neurodegenerative disorders is mainly from the studies with children and young adults in Mexico City [66,67,68]. Overall, these studies demonstrated that those children presented early markers of neurodegeneration, neuroinflammation (e.g., elevated macrophage inhibitory factor (MIF), IL-6, and IL-2 in CSF [69] and increased IL-6 and Toll-like receptors (TLRs) expression in frontal cortex), olfactory dysfunction, and cognitive deficits compared to control children from nearby non-polluted cities. Of these biomarkers examined, MIF is a cytokine essential for microglial activation and the production of IL-6, IL-1β, TNF-α, and iNOS [70].

More recently, the association of PM_2.5_-associated microglial activation with neurodegenerative disorders have been investigated among the elderly. In a panel study, Tang et al. examined the air pollutant (including PM) exposure in Chinese people aged 60–69 years, showing that air pollution exposure could induce alterations of neurodegenerative biomarkers, such as Aβ_40_, Aβ_42_, α-synuclein, PrPC, Tau (pThr181), and the activation of microglia represented by the over-expression of S100B and microglial triggering receptor expressed on myeloid cells2 (TREM2) [71]. Our recent findings from a panel study on healthy retired adults demonstrated that PM_2.5_ concentration increments were associated with increases in the biomarkers of neural damage, including a neurofilament light chain (NfL), neuron-specific enolase, and the activation of microglia (S100B) in serum [72]. Meanwhile, several constituents of ambient PM_2.5_, such as Cu, Zn, Ni, Mn, Sn, V, Rb, Pb, Al, Be, Cs, Co, Th, U, Cl^−^, and F^−^ were found to be significantly associated with serum levels of NfL [72].

In summary, the available evidence in humans, albeit limited and variable, is suggestive of the association of PM_2.5_ exposure with neurodegenerative disorders.

**Table 1:** Major evidence from epidemiological investigations.

Study Design	Location	Subjects	Exposure	Outcome	Results	References
Cohort	Taiwan, China	95,690 individuals’ age ≥ 65	PM_2.5_, PM_10_ and O_3_	Newly diagnosed AD in Taiwan from 2001–2010	A 138% risk of increase of AD per increase of 4.34 g/m^3^ in PM_2.5_ over the follow-up period (95% CI: 2.21–2.56).	Jung et al. [52]
Cohort	the Ruhr area and Southern Muensterland, Germany	789 women	Air pollution (including PM_2.5_)	Cognitive performance and function	PM_2.5_ was negatively associated with cognitive function and cognitive performance (β = −0.19 (95% CI: −0.36 to −0.02)).	Schikowski et al. [53]
Cohort	Ontario, Canada	4.4 million adults for a multiple sclerosis cohort; 2.2 million adults for dementia or Parkinson’s disease cohort	Traffic-related air pollution (including PM_2.5_)	Residential proximity to roads; Incidence of multiple sclerosis, dementia, and Parkinson’s disease.	The incidence of dementia was associated with the distance to roads: (HR = 1.07, 95% CI: 1.06–1.08) for <50 m; (HR = 1.04, 95% CI: 1.02–1.05) for 50–100 m; (HR = 1.02, 95% CI: 1.01–1.03) for 101–200 m; (HR = 1.00, 95% CI: 0.99–1.01) for 201–300 m.	Chen et al. [54]
Case-crossover	Communities from different sites in the USA	Medicare enrollees (>65 years)	PM_2.5_	The risk of hospitalization for neurological disorders; The association between short-term exposure to PM_2.5_ and all-cause mortality.	Increased hospitalization risks for Parkinson’s disease (3.23% increase, 95% CI: 1.08–5.43) for a 10 μg/m^3^ increase in the 2 days average.	Zanobetti et al. [55]
Cohort	50 northeastern U.S. cities	9.8 million Medicare enrollees (≥65 years)	PM_2.5_	Time to first hospitalization for dementia, Alzheimer’s, or Parkinson’s diseases.	Per 1-μg/m^3^ increase in annual PM_2.5_ concentrations: HR of 1.08 (95% CI: 1.05–1.11) for dementia; HR of 1.15 (95% CI: 1.11–1.19) for AD; HR of 1.08 (95% CI: 1.04–1.12) for PD admissions	Kioumourtzoglou et al. [56]
prospective pilot study	Mexico City metropolitan area (MCMA) and small cities with clean air for control	129 children and adults	PM_2.5_	Neurodegenerative biomarkers in CSF: Aβ_42_, α-synuclein (t-α-syn and d-α-synuclein).	Decreased levels of Aβ_42_ and BDNF in MCMA children (*p* = 0.005 and 0.02, respectively). Total synuclein showed an PM_2.5_-dependent increase and then a decrease after age 12 years, while d-α-synuclein exhibited a tendency to increase with cumulated PM_2.5_ (R^2^ = 0.30).	Calderón-Garcidueñaset al. [57]
Prospective pilot stud	MCMA and small cities with clean air for control	507 healthy children and adults	High vs. low air pollution	Non-phosphorylated tau(non-P-Tau) and Aβ_42_ in the cerebrospinal fluid.	A strong increase in Non-P-Tau with age, which was faster among MCMA children versus controls (*p* = 0.0055). Aβ_42_ and BDNF concentrations were lower in MMC children (*p* = 0.002 and 0.03, respectively).	Calderón-Garcidueñaset al. [59]
Prospective cohort study	Communities from different sites in the USA	1403 community-dwelling older women (71–89 years)	PM_2.5_	Volume of gray matter (GM) and normal-appearing white matter (WM).	Older women with greater PM_2.5_ exposures had significantly smaller WM. A 4.47 cm^3^ decrease (95% CI: 2.27–6.67) in the volume of WM per increase of 3.49 μg/m^3^ in PM_2.5_.	Chen et al. [61]
Prospective cohort study	48 states of the USA	998 older females aged (73–87 years)	PM_2.5_	Tests of immediate free recall/new learning (List A Trials 1–3; List B) and delayed free recall (short- and long-delay).	PM_2.5_ was associated with greater declines in immediate recall and new learning: the annual decline rate was significantly accelerated by 19.3% (95% CI: 1.9–36.2%) for Trials 1–3 and 14.8% (95% CI: 4.4–24.9%) for List B per increase of 3.49 μg/m^3^ in PM_2.5_.	Younan et al. [62]
Cohort	North Carolina and Iowa of the USA	84,739 farmers	PM_2.5_ and O_3_	The incidence of Parkinson’s disease.	A positive association of Parkinson’s disease with PM_2.5_ (OR = 1.34; 95% CI: 0.93–1.93) in North Carolina but not in Iowa.	Kirrane et al. [63]
Nested case-control	Different states of the USA	1556 Parkinson’s disease cases and 3313 controls	PM_2.5_, PM_10_ and NO_2_	The incidence of Parkinson’s disease.	A higher risk of PD was associated with higher exposure to PM_2.5_ (OR_Q5 vs. Q1_ = 1.29; 95% CI: 0.94–1.76; *p* = 0.04) among non-smokers.	Liu et al. [64]
Cohort		115,767 healthy women	PM_2.5_ and PM_10_	The incidence of Parkinson’s disease.	No statistically significant associations between PM_2.5_ exposure and PD risk (RR = 1.08, 95% CI: 0.81–1.45).	Palacios et al. [65]
Panel	Jinan, China	76 people aged 60–69 years	High level of air pollution (including particulate matters)	Neurodegenerative biomarkers: Aβ_40_, Aβ_42_, α-synuclein, PRNT, Tau(pThr181); Activation of microglia: S100B, TREM2).	Air pollution exposure induces the alterations of neurodegenerative biomarkers, such as Aβ_40_, Aβ_42_, α-synuclein, PRNP, Tau (pThr181), and the activation of microglia.	Tang et al. [71]
Panel	Xinxiang, Chian	34 healthy retirees from Xinxiang Medical University	PM_2.5_	Biomarkers of neural damage in serum: NfL, NSE, PGP9.5, S100B.	PM_2.5_ and its key constituents were significantly associated with neural damage biomarkers: A 10 μg/m^3^ increase in PM_2.5_ concentration was associated with 2.09% (95% CI: 39.3–76.5%), 100% (95% CI: 1.73–198%), and 122% (95% CI: 20.7–222%) increments in BDNF, NfL, and PGP9.5, respectively. Several constituents such as Cu, Zn, Ni, Mn, Sn, V, Rb, Pb, Al, Be, Cs, Co, Th, U, Cl^−^, and F^−^ were significantly associated with NfL.	Song et al. [72]

## 5. Evidence from Animal Studies

In vivo studies in general corroborate and expand the major pathophysiological findings in human brain tissue and other accessible tissues [73,74], such as the markers of oxidative stress, neuroinflammation, and neurodegeneration, and help decipher underlying mechanisms linking exposure to the development of neurodegenerative disorders. Most of the animal studies have focused on the effects of PM_2.5_, especially DEP and UFPM, on AD-like pathologies (see Table 2 for details). 

In AD condition [75], microglia-mediated neuroinflammation is a critical event characterized by the release of IL-1β, IL-6, and TNF-α. This feature has been verified in animals exposed to PM_2.5_ under diverse exposure scenarios, mostly chronic exposure. For example, children and dogs chronically exposed to severely polluted air pollution in Mexico City displayed similar inflammatory neuropathological lesions [10,76]. The chronic inhalation of airborne PM_2.5_ caused time-dependent early AD-like changes in mice, such as an increase in Aβ_40_, BACE (beta-site amyloid precursor protein (APP)-cleaving enzyme), and COX-2, as well as a decrease in APP, with a minimal change of phosphorylated tau [77]. The oropharyngeal aspiration of PM_2.5_ for 4 weeks induced a dose-dependent increase in IL-1β and TNF-α in the blood and hippocampus of mice [78]. The whole-body exposure of rats to PM_1.0_ for 3 and 6 months resulted in microglia activation in the hippocampus [79]. In addition, short-term exposure showed similar neurological effects. For example, mice exposed for 5 d to a traffic-polluted highway tunnel exhibited increased expression of microglia-associated inflammatory genes (COX-2, iNOS, and nuclear factor) in the hippocampus, and decreased BDNF expression in the OB [80]. 

DEP are a major constituent of ambient PM_2.5_ and are commonly used as a surrogate model of air pollution in health effects studies [81]. The short- or long-term inhalation of DEP can induce the over-expression of pro-inflammatory factors in select brain regions [82,83]. For example, the inhalation of DEP (0.5 and 2 mg/m^3^, for 1 month) increased IL-1β, TNF-α, IL-6, MIP-1α (macrophage inflammatory protein-1α), fractalkine, and Iba-1 in most regions of Sprague Dawley (SD) rats, with the midbrain showing the greatest DEP response [84]. Meanwhile, a single intratracheal administration of DEP increased microglial Iba-1 levels in the substantia nigra and elevated serum and whole-brain TNF-α at 6 h post-treatment [85]. The susceptibility of the midbrain to DEP neuroinflammatory effects was confirmed by another inhalation study over 6 months on male Fischer 344 rats exposed to DEP (35–992 μg/m^3^), probably due to the most microglia in the midbrain [85]. In addition, TRAP-related PM has also been examined for neurological effects. For example, SD rats exposed to PM_1_ (250–300 μg/m^3^) for 3 and 6 months demonstrated that PM_1_ induced cytotoxicity, lipid peroxidation, microglial activation, and inflammation as well as autophagy and caspase-3 up-regulation in microglia [80]. Wistar rats inhaling DEP nanoPM (0.3–1.0 mg/L) for 3 months had higher levels of COX-2 and Aβ_42_ in brain regions [86]. An interesting finding came from transgenic mice with human APOE3 and E4 alleles, showing that chronic exposure to nanoPM over 15 weeks increased the cerebral Aβ production and deterioration of hippocampal CA1 neurons, with a more significant effect in APOE4 carriers [87]. 

The role of microglia in DEP-induced neurodegenerative disorders has been explored mostly in mouse models. The acute exposure of adult mice to DEP (250–300 mg/m^3^ for 6 h) caused microglial activation, lipid peroxidation, and reduced neurogenesis in all brain regions, particularly in the hippocampus and the OB [88]. The blockage of microglial activity with the PPAR-γ agonist pioglitazone inhibited the DEP-induced neuroinflammation in cerebral cortex, oxidative stress, and neurogenesis reduction in the hippocampus [89]. The exposure of mice to TRAP-PM_0.2_ (300 μg/m^3^) or of neuronal cells to the same nanoPM (1–10 μg/mL) caused an increase in oxidative stress in lipid rafts associated with an increase in Aβ, the latter was inhibited by the antioxidant N-acetyl cysteine, suggesting that oxidative stress was involved in the pro-amyloidogenic effects of air pollution [90]. Interestingly, Cheng et al. examined the differential time course of oxidative stress and inflammatory responses to UFPM between the OE and the brain. It was found that OE and OB, but not the cerebral cortex and cerebellum, had rapid increases in microglial number, and oxidative and nitrosative protein adducts in the nasal epithelium turbinate after 5 h exposure, which precedes an increase in levels of TNF-α by 45 h [91]. These responses corresponded to in vitro OE and mixed glial responses, with the rapid induction of nitrite and iNOS preceding the induction of TNF-α [91] Furthermore, wild-type (WT) and *Nrf2* knockout (*Nrf2*^−/−^) mice were subjected to the intranasal instillation of 1 mg/kg PM_2.5_ for 28 days. Lower levels of antioxidant enzymes, oxidative stress, microglia activation, inflammation, NF-κB activation, and severe nerve injury were detected in the OB of Nrf2^−/−^ mice compared to the OB of WT mice [92]. In addition, PM_2.5_ exposure-induced oxidative stress and microglia activation was attributed to its metal contents and glutaminase-containing extracellular vehicles (EVs) in the OB [93].

**Table 2:** Evidence from main animal studies with PM_2.5_.

Animal	Exposure Protocol	Pathological Changes	Conclusion	References
Male C57BL/6 mice (8 weeks)	PM_2.5_: 6 h/day, 5 days/week, for 3 and 9 months (65.7 ± 34.2 μg/m^3^). Filtered air for controls.	9 months: increased COX-1, COX-2, APP, BACE, Aβ_1–40_, PSD-95 and cytokines levels. 3 months: no difference of all these biomarkers.	Long-term exposure to high dose PM_2.5_ could alter brain inflammatory phenotype, induce synapse damage and promote AD-like pathology.	Bhatt et al. [77]
Male C57BL/6 mice (8 weeks)	Oropharyngeal aspiration of PM_2.5_ (1 and 5 mg/kg bw) every other day for 4 weeks. Saline for controls.	A dose-dependent increase in IL-1β and TNF-α in the blood and hippocampus. Increased BACE1 (biomarker of synaptic function) expression.	Chronic exposure to PM_2.5_ causes neuroinflammation, deteriorated synaptic function integrity.	Ku et al. [78]
Male SD rats (6 months)	Traffic-related PM_1_ (aerodynamic diameter < 1 μm): 6 h/day, for 3 and 6 months (16.3 ± 8.2 μg/m^3^) Filtered air for controls.	Elevated levels of TBARSs, PGE2, TNF-α and Iba-1.	Traffic-related PM exposure causes microglia activation, neuroinflammation and oxidative stress in the brain.	Bai et al. [79]
C57BL/6 mice (6 weeks)	Traffic-polluted highway tunnel for 5 days (mean PM _2.5_ 55.1 μg/m^3^, mean elemental carbon 13.9 μg/m^3^). Filtered air for controls.	Increases in COX-2, NOS2, and NOS3 genes (encoding the COX-2, iNOS, and eNOS, respectively) in the hippocampus. Decreased level of BDNF in the olfactory bulb.	Short-term exposure to traffic-related air pollution induces the differential expression of inflammatory and oxidative genes in different brain regions. The olfactory bulb may display a lower neurotrophic support in response to air pollution.	Bos et al. [80]
SD rats (12 weeks)	DEP: 4 h/day, 5 days/week, for 1 month (0.5 or 2 mg/m^3^) and 0 mg/m^3^ for controls.	Elevated levels of whole-brain IL-6, nitrated proteins and Iba-1 (biomarker of microglia activation). The midbrain displayed a higher sensibility to DEP.	Inhalation of DEP causes various degrees of microglia activation and neuroinflammation in different brain regions.	Levesque et al. [84]
Male Fischer 344 rats (10–12 weeks)	DEP: 6 h/day, 7 days/week, for 6 months (35, 100, 311 and 992 μg/m^3^) Filtered air for controls.	Elevated level of TNF-α at high concentrations (most at 992 μg/m^3^) in all regions, with the exception of the cerebellum. Increased level of TNF-α at 100 μg/m^3^ midbrain.	The midbrain may be more sensitive to the neuroinflammatory effects of DEP exposure.	Levesque et al. [85]
Female EFAD transgenic mice (E3FAD, E4FAD at 3 months)	nPM: 5 h/day, 3 days/week, for 15 weeks (10 μg/mL). Filtered air for controls.	In both genotypes: increased levels of Aβ generation and deposition in the cerebral and CA1 neurites atrophy, decreased glutamate GluR1 subunit level. E4FAD mice displayed more significant neurotoxicity of nPM.	Long-term nPM exposure could promote the generation and accumulation of Aβ and the neuronal damage, which further leads to neurodegeneration.	Cacciottolo et al. [87]
Adult mice (both sexes at 8 weeks)	DEP: 250–300 μg/m^3^ for 6 h. Filtered air for controls.	Increased levels of IL-1β, TNF-α and MDA in all brain regions, especially the OB and hippocampus. Decreased level of BrdU in the hippocampus. Male mice showed higher increase in IL-1β, TNF-α, MDA levels.	Acute exposure to DEP may cause neurotoxicity (neuroinflammation, oxidative stress, and neurodegeneration). Males may be more sensitive to the neurotoxicity of DEP.	Costa et al. [88]
C57BL/6J mice (both sexes at 8 weeks)	DEP: 250–300 μg/m^3^ for 6 h. Filtered air for controls.	Decreased numbers of new neurons in the SGZ, SVZ, and OB, while only in the OB in females. Elevated numbers of activated microglia and the levels of TNF-α and MDA in the cortex and hippocampus, which was decreased by pioglitazone treatment.	Acute DEP exposure leads to neuroinflammation, oxidative stress and disordered neurons genesis, which was more severe in males and seems to be associated with the activation of microglia.	Coburn et al. [89]
Male C57BL/6J mice (3 months)	nPM: for 5, 20, and 45 h over 3 weeks. Filtered air for controls.	Rapid increases of 4-HNE and 3-NT protein in OB and OE at 5 h. Increased numbers of microglia in OB and nasal epithelium turbinate. Elevated level of TNF-α in all brain regions at 45 h, with an earlier increased level of TNF-α mRNA in the OB and OE.	Acute nPM exposure could induce the activation of microglia, neuroinflammation, and oxidative stress in different brain regions, especially the OE and OB.	Cheng et al. [91]
Male C57BL/6 and Nrf2^−/−^ mice	Intranasal instillation of PM_2.5_ for 28 days (1 mg/kg bw). Deionized water for controls.	Decreased levels of antioxidant enzymes (GSH, SOD) and increased levels of MAD, inflammatory cytokines, and activation of microglia and NFκB in the OB. Increased neuron apoptosis in the olfactory bulb.	Nrf2 may play a neuroprotective role in response to PM_2.5_ exposure.	Chen et al. [92]
Male C57BL/6 mice (6 weeks)	Daily intranasal instillation of PM_2.5_ (0.1 or 1 mg/kg bw), Chelex-treated PM_2.5_ (1 mg/kg bw), PM_2.5_ (1 mg/kg bw) plus CB-839 (glutaminase inhibitor) for 28 days. Deionized water for controls.	Elevated levels of ROS generation, microglia activation, EVs release, and GAC expression in the OB. Treated with CB-839 significantly decreased the number of EVs and the expression of GAC.	PM_2.5_ exposure could activate microglia and may mediate its neurotoxicity by promoting the production of glutaminase-containing EVs.	Chen et al. [93]

Abbreviations: COX-1, cyclooxygenase-1; COX-2, cyclooxygenase-2; APP, amyloid precursor protein; BACE, beta-site APP cleaving enzyme; PSD-95, pre- and post- synaptic marker; nPM, nanosized particulate matter; DEP, diesel exhaust particle; Iba-1, ionized calcium-binding adaptor molecule 1; TBARSs, thiobarbituric acid-reactive substances; PGE2, prostaglandin E2; NTS, nucleus of solitary tract; MDA, malondialdehyde; ROS, reactive oxygen species; OB, olfactory bulb; BrdU, brmodeoxyuridine; SGZ, hippocampal subgranular zone; SVZ, the subventricular zone; 4-HNE, 4-hydroxy-2-nonenal; 3-NT, 3-nitrotyrosine; OE, olfactory neuroepithelium; iNOS, inducible nitric oxide synthase; eNOS, endothelial nitric oxide synthase; EVs, extracellular vesicles; GAC, glutaminase C.

In PD condition, α-synuclein is a major component of Lewy bodies, a pathological hallmark of PD [94]. A controlled study found that exposure of male Fischer 344 rats to DEP (311 μg/m^3^ or higher) for 6 months increased α-synuclein and Aβ_42_ levels in the midbrain [85]. In another study, an increase in α-synuclein levels was also found in the cerebral cortex of C57BL6/J mice exposed to DEP (250 μg/m^3^) for 3 weeks [1].

In summary, animals exposed to PM ambiently or in controlled experiments reveals the same pattern of neurotoxic effects as in humans although the crosstalk among these events need further clarification.

## 6. Evidence from In Vitro Studies

In vitro studies provide insights into in-depth cellular and molecular mechanisms by which PM exposure promotes cellular damage and abnormality, linking to neurodegenerative disorders, such as the alterations of cell viability and apoptosis, the dysfunction of mitochondria, the production of ROS, or the release of pro-inflammatory mediators [1,17]. Thus far, neuronal and microglial cell lines, primary cultures or co-culture of those cells have been introduced for exposure to concentrated ambient air particles, DEP, and LPS, among others [95] (Table 3). 

A mouse microglial cell line (BV2) exposed to concentrated ambient PM_2.5_ displayed the upregulated mRNA of pro-inflammatory cytokines, such as IL-1β and TNF-α [96]. Moreover, the inhibition of Nrf2 activity significantly blocked the PM_2.5_-induced decrease in cell viability, the increase in the intracellular ROS generation, and the NFκB phosphorylation in BV2 cells [92]. The acute exposure of microglial cells to high-dose PM_2.5_ decreased cell survival as a result of neuroinflammation and the production of ROS [97,98]. In addition, an in vitro neuron-microglia culture model exposed to PM_2.5_ presented elevated apoptosis, IL-1β, and caspase-1 activity, which could be alleviated by the addition of IL-1 receptor antagonists and ROS inhibitors [98]. Together, these findings suggest that PM_2.5_ has a role in AD pathogenesis, the underlying mechanisms possibly being PM_2.5_-induced microglial activation, neuroinflammation, increased ROS activity, and even Aβ production [95]. Notably, there is evidence that metals associated with PM_2.5_ may activate microglia, since microglia can be activated in vitro by manganese [99]. 

Microglia were first shown to recognize and respond to PM in an in vitro study using DEP [100]. Generally, in vitro studies support in vivo observations by showing that DEP can activate microglia, resulting in oxidative stress and neuroinflammation [86,100,101]. With BV2 cells, DEP were shown to reduce cell viability and increase microglial activation, lipid peroxidation, the production of pro-inflammatory mediators, including IL-6, TNF-α and prosglandin E2 (PGE2), and cytotoxicity [80]. Intriguingly, exposure to DEP (25–100 μg/cm^2^) did not affect the viability of mouse primary cerebellar granule neurons in vitro. However, the death of these neurons was increased two- or three-fold with simultaneous exposure to DEP and microglia [101]), suggesting that microglia play an essential role in DEP-induced neurotoxicity.

**Table 3:** Evidence from main in vitro studies with PM_2.5_.

Cell Type	Species	Exposure Protocol	Pathological Changes	Conclusion	References
Microglia cell line (BV2)	Mouse	DEP: 50 and 100 μg/mL for 24 h. 0 μg/mL for control.	Increased levels of ROS, LDH, TBARSs, IL-6, PGE2, and TNF-α and decreased cell viability. Microglia activation.	Acute exposure to DEP could induce cytotoxicity, lipid peroxidation, microglial activation and inflammation.	Bai et al. [79]
Microglia cell line (HAPI) and primary neurons	Rat	DEP: 5–50 μg/mL for 3 and 24 h. 0 μg/mL for control.	Increased levels of NO, TNF-α and DA injury (5 μg/mL group) and H_2_O_2_ generation in microglia co-treatment with LPS (2.5 ng/mL).	DEP exposure causes neuroinflammation, oxidative stress and neuron death, which may be associated with the activation of microglia.	Levesque et al. [84]
Microglia cell line (BV2)	Murine	PM_2.5_: 50 μg/mL for 24 h. 0 μg/mL for control.	Decreased cell viability and increased intracellular ROS generation and NF-κB phosphorylation when the Nrf2 activity was inhibited.	Nrf2 may play anti-oxidation and anti-inflammation roles in response to PM_2.5_ exposure in the neurons.	Chen et al. [93]
Microglia cell line (BV2)	Mouse	CAPs (≤2.5 μm): 75 μg/mL for 4 h and 25–100 μg/mL for 1.5 h or 6 h. 0 μg/mL for control.	Decreased levels of intracellular ATP (≥250 mg/mL) and depolarized mitochondrial membranes (≥6 mg/mL). Release of pro-inflammatory cytokines (TNF-α and IL-6). Up-regulated expression of inflammatory genes.	CAPs exposure could induce an inflammatory response and regulate the gene expression in BV2, and the mitochondrial injury may be key to CAPs-induced neurotoxicity.	Sama et al. [96]
Microglia cell line (BV2)	Rat	PM_2.5_: 5, 10, 25, 50, 100 μg/mL for 1 h and 24 h. 0 μg/mL for control.	Increased levels of NO and ROS generation and the genes expression of IL-1β, IL-6, COX-2, and iNOS, especially in high dose groups. Microglia activation of M1 phenotype. Decreased cell viability.	Acute PM_2.5_ exposure probably mediates its neurotoxicity through inflammation and oxidative stress in the microglia.	Kim et al. [97]
Primary microglial cells and neurons	mouse	PM_2.5_: 50 μg/mL for 4 h. 0 μg/mL for control.	Elevated levels of IL-1β, caspase-1 activation and ROS generation. Inhibition of IL-1 receptor and ROS generation decreased the levels of inflammatory cytokines and cell apoptosis.	Acute PM_2.5_ exposure would cause neuroinflammation and oxidative stress, which may induce neurons apoptosis.	Wang et al. [98]
Microglia cell line (HAPI) and primary microglial	Rat	MnCl_2_: 0.33, 1, 3.33, 10, 33 μM for 0.25, 1, 3, 6 and 24 h. 0 μM for control.	An increased time- and concentration-dependent release of hydrogen peroxide (H_2_O_2_) in microglia.	MnCl_2_ is capable of activating microglia to release ROS.	Zhang et al. [99]
Primary microglial cells and neurons	Rat	DEP (0.22 μm): 5–50 μg/mL. 0 μg/mL for control.	Dose-dependent microglia activation. Selective dopaminergic neuron (DA) death induced by DEP treatment was reinstated with the addition of microglia. Microglia from mice missing functional NADPH oxidase displayed insensitive response to DEP treatment.	Microglia may play a key role in DEP-induced neurotoxicity.	Block et al. [100]
Primary microglia cells and cerebellar granule neurons (CGNs)	Mouse	DEP: 25, 50, 100 μg/2 cm^2^ for 24 h. 0 μg/2 cm^2^ for control.	DEP treatment did not affect the viability of CGNs. Neuronal cell death increased by 2–3-fold after co-treatment with microglia. Elevated level of ROS genes expression of IL-1β and IL-6 in microglia.	Microglia may mediate DEP-induced neuronal toxicity through oxidative stress and neuroinflammatory mechanisms.	Roqué et al. [101]

Abbreviations: CAPs, concentrated ambient particles; ROS, reactive oxygen species; NF-κB, nuclear factor kappa B; iNOS, inducible nitric oxide synthase; DEP, diesel exhaust particle; HAPI, highly aggressively proliferating immortalized; TBARSs, thiobarbituric acid-reactive substances; PGE2, prostaglandin E2.

In the PD condition, primary neuron–glia co-cultures and the HAPI (highly aggressively proliferating immortalized) microglial cell line were pre-treated with DEP (5 μg/mL) followed by LPS (2.5 ng/mL) and synergistically amplified NO production, TNF-α release, and DA neurotoxicity [84]. Pre-treatment with fractalkine (50 pg/mL), a chemokine from neurons as a soluble anti-inflammatory signal for microglia [102], ameliorated DEP (50 μg/mL)-induced H_2_O_2_ production from microglia and protected against DEP-induced DA neurotoxicity in midbrain neuron–glia cultures [84]. Another study with mesencephalic neuron–glia cultures treated with DEP (5–50 μg/mL) resulted in a dose-dependent microglial activation determined by changes in morphology and increase in superoxide production and a decrease in DA neurons, with no TNF-α, NO, or prostaglandin PGE2 detected [100]. Noticeably, the selective DA neurotoxicity only occurred in the presence of microglia, indicating that microglia mediated the neuron damage. In addition, a study revealed that microglia cultures derived from mice missing functional NADPH oxidase, the enzyme responsible for microglial extracellular superoxide production, were insensitive to DEP-induced neurotoxicity, indicating that microglia-derived ROS are key for DEP-induced DA neurotoxicity [100]. Thus, it is assumed that the neurotoxic effects of DEP on DA neurons could be either direct or indirect via the release of inflammatory mediators and ROS from activated microglial cells [84,100,102]. 

Overall, in vitro studies suggest PM-induced oxidative stress and microglial cell mediated inflammatory and/or oxidative responses as potential mechanisms leading to neurotoxicity, and the increased risk of neurodegenerative disease as seen in epidemiological and animal studies. 

## 7. Potential Mechanisms for PM_2.5_-Induced Microglial Oxidative Stress and Neuronal Toxicity

The aforementioned evidence suggests that exposure to PM_2.5_ has a crucial role in neurodegenerative disorders possible through the activation of microglial cells [95]. However, the responsible mechanisms remain large unknown. The four critical issues are of significant relevance and need to be addressed: (1) The routes through which PM_2.5_ access CNS; (2) the receptors microglia use to relay PM_2.5_ neurotoxic signals; (3) PM_2.5_-induced microglial oxidative stress; and (4) the interactions between microglia and neurons. 

**Figure 2:**
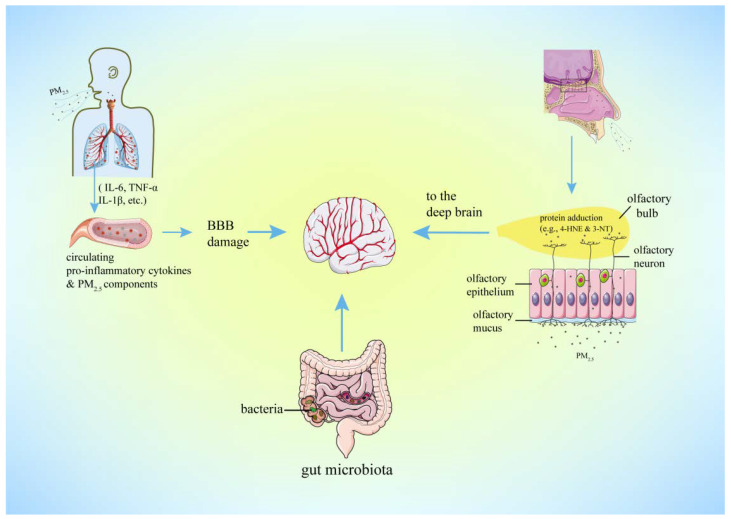
The routes that PM_2.5_ enters the brain. The lung–brain axis and olfactory pathway are two recognized predominant routes that PM_2.5_ takes into the brain. Once inhaled, PM_2.5_ can quickly diffuse throughout the alveoli and lead to lung inflammation. These circulating cytokines (IL-6, TNF-α, IL-1β, etc.), in combination with soluble components of PM_2.5_, cross the BBB directly or via a disruption to the permeability of the BBB, and then induce microglia activation and neurotoxicity. Meanwhile, with a consequence of lipid peroxidation (4-HNE and 3-NT protein adduction), PM_2.5_ could also gain access to the olfactory bulb through the olfactory epithelium and then move into the deep regions of the brain. Moreover, the gut–brain axis is potentially another route through which PM_2.5_ exerts its neurotoxicity, which is probably associated with the dysbiosis of gut microbiota.

**Route of CNS Effects**: Multiple routes for PM_2.5_ impact on the CNS have been proposed, two of them are predominant (Figure 2). First, PM_2.5_, UFPM, and their components can enter the olfactory receptor neurons that extend their dendrites into the mucous layer covering the OE through pinocytosis, simple diffusion, or receptor-mediated endocytosis, and is further transported along the axons to the OB and olfactory cortex [9,103]. In addition, UFPM exposure has been shown to rapidly increase the products of lipid peroxidation, such as 4-hydroxy-2-nonenal (4-HNE) and 3-nitrotyrosine (3-NT) protein adducts in the OE and OB [91], further leading to oxidative inflammation from nose to brain. Second, PM_2.5_ inhaled into the deep lung successively penetrates the alveolar–blood barrier and the BBB and finally reaches brain regions. Meanwhile, lung-derived circulating cytokines induced by PM_2.5_ exposure could also enter the brain [18]. In both cases, PM_2.5_ directly or indirectly activates microglia and induces the release of pro-inflammatory cytokines and ROS, leading to neurodegeneration [66,104]. Alternatively, PM_2.5_ may potentially affect the CNS via the gut microbiota–brain axis [1,105] or lung microbiota [106]. 

**Figure 3:**
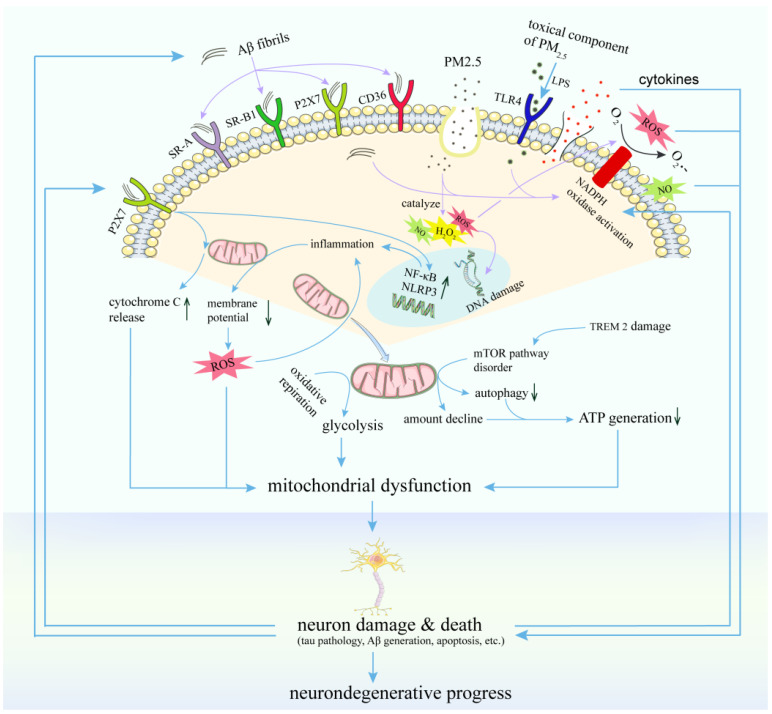
Proposed mechanisms for PRR-mediated microglia activation and neuronal toxicity induced by PM_2.5_. Microglia monitor the brain environment by interpreting and processing stimuli through pattern recognition receptors (PRRs), which mainly include TLRs, scavenger receptors, MAC1, and receptor complex for diverse neurotoxic and pro-inflammatory ligands, respectively. Exogenous and endogenous insults bind to diverse PPRs and result in microglial activation and release of soluble factors, such as cytokines, PGE2, and neurotrophins (BDNF), which bind to neuronal receptors. Meanwhile, neuronal metabolites and damaged neuron components could also activate microglia. Together, microglia-neuron interactions further promote the pathogenesis of neurodegenerative disorders.

**Sensing receptors on microglia**: Microglia monitor the brain environment by interpreting and processing stimuli through pattern recognition receptors (PRRs) (Figure 3), which mainly include TLRs [107,108], scavenger receptors (e.g., SR-A1 and SR-B1) [109], macrophage antigen complex 1 (MAC1), and receptor complexes (CD36, α6β1 integrin, and CD47) [16], for diverse neurotoxic and pro-inflammatory ligands, respectively. Both nanoPM and LPS have been shown to strongly activate TLR4 and NF-κB in mixed glial cultures. TLR4 siRNA attenuated TNF-α and other inflammatory responses to nanoPM via the MyD88-dependent pathway [110]. Thus, PPRs expressed on the microglial surface seem to be one of the primary common pathways by which ambient PM signals are transduced into ROS production [16].

**Microglia-associated oxidative stress:** ROS are critical components of the pro-inflammatory signaling pathway in microglia [110]. Activated microglia by exogenous and endogenous insults can become a chronic source of pro-inflammatory factors and oxidative stress in the brain, driving neurodegenerative diseases [16]. In microglia, ROS primarily from both NOX and the mitochondria, may act as second messengers to propagate immune activation, excessive inflammation, and oxidative stress [111]. Ambient particles transported into the brain could be phagocytized by microglia, leading to NOX and microglial activation, and ROS production [84]. Mitochondrial dysfunction in microglia has been proposed to play a role in the progression of neurodegenerative diseases [112]. The elevated generation of ROS and the loss of mitochondrial membrane potential through various mechanisms have been observed in AD. Aβ interacts with microglial receptors, such as TREM2, activating downstream pathways, causing mitochondrial damage, and aggravating inflammation and cytotoxicity. Fibrillar Aβ activates NOX in microglia leading to the elevated induction of mitochondrial ROS, which further causes neurotoxicity [112]. 

**Microglia-neuron interactions:** The bi-directional communication between microglia and neurons has been recognized to be critical for maintaining a healthy environment in the CNS and also for the chronic development of neuroinflammation [113]. The air pollution-induced loss of neurons has been detected in postmortem and experimental studies, as described earlier, and neuronal cell death may be direct or indirect via microglia activation [12]. Thus far, the mechanisms for microglia–neuron interaction remain elusive. Activated microglia can release soluble factors, such as cytokines (IL-1β; TNF-α), PGE2, and neurotrophins (BDNF), which bind to neuronal receptors [114]. With a primary cerebellar granule neuron (CGN) model, DEP showed minimal effect on neuron viability after 24 h of treatment. In the presence of primary cortical microglia neuronal cell death increased by 2–3 fold after co-treatment with DEP, suggesting that microglia are important contributors to DEP-induced CGN neurotoxicity, possibly due to soluble intermediates since microglia-conditioned medium by DEP treatment was also toxic to CGNs [101]. In addition, Block et al. showed that DEP could damage DA neurons through microglia-derived oxidant species [104]. However, another study reported that DEP caused a significant increase in ROS in microglia, antioxidants failed to protect neurons from DEP/microglia-induced toxicity [105]. From the above, the mechanisms underlying PM_2.5_-induced microglial activation and its interaction with neurons are still unclear and warrant further investigation. 

## 8. Conclusions and Future Directions

In summary, air pollution, together with the increasing age of the global population, pose great threats to public health. Thus far, the mechanisms responsible for PM_2.5_-induced neurodegenerative diseases remain largely unknown. The CNS effects are chronic, beginning in childhood, and may take time to accumulate pathology. Specifically, air pollution has been shown to cause neuroinflammation, oxidative stress, cerebral vascular damage, and neurodegenerative pathology, which all involve microglial activation. Evidence from epidemiological and experimental studies suggests that exposure to ambient PM, especially PM_2.5_, is associated with neurodegenerative disorders. The interpretation of the intracellular and extracellular pathways participating in the generation of oxidative stress in microglia may be important not only for comprehending the pathophysiological basis for neuron damage in neurodegenerative diseases but also for designing effective strategies to mitigate or even prevent PM_2.5_-induced neural neurodegenerative damage. While epidemiology has linked an increased risk of stroke, AD, and PD with exposure to PM_2.5_, further epidemiological and mechanistic studies regarding the association between the components of air pollution and the development of CNS diseases are of pressing concern for human health. 

## Abbreviations

PM_2.5_, PM with a diameter less than 2.5 μm; UFPM, ultrafine PM; CNS, central nervous system; TRAP, traffic-related air pollution; DEP, diesel exhaust particles; LPS, lipopolysaccharides; AD, Alzheimer’s disease; PD, Parkinson’s disease; ROS, reactive oxygen species; RNS, nitric oxide species; IFN-γ, interferon-γ; Aβ, amyloid β; TNF-α, tumor necrosis factor-α, IL-6, interleukin 6; IL-1β, interleukin 1β; IL-12, interleukin 12; CCL-2, chemokine ligand-2; MCP-1, monocyte chemoattractant protein-1; PGE, prosglandin E; NADPH, nicotinamide adenine dinucleotide phosphate; NOX, nicotinamide adenine dinucleotide phosphate oxidase; iNOS, inducible nitric oxide synthase; NO, nitric oxide; Iba-1, ionized calcium binding adapter molecule-1; PPARγ, peroxisome proliferator-activated receptors γ; TGF-β, transforming growth factor-β; CSF-1, gcolony stimulating factor-1; BDNF, brain derived neurotrophic factor; NF-κB, nuclear factor-κB; BBB, blood-brain barrier; NFTs, neurofibrillary tangles; OE, olfactory epithelium; OB, olfactory bulbs; PrPC, cellular prion protein; GFAP, glial fibrillary acidic protein; APOE4, Apolipoprotein E4; APOE3, Apolipoprotein E3; COX-2, cyclooxygenase-2; 8-OHdG, 8-hydroxydeoxyguanosine; CSF, cerebrospinal fluids; MIF, macrophage inhibitory factor; TLRs, Toll-like receptors; TREM2, triggering receptor expressed on myeloid cells 2; NfL, neurofilament light chain; BACE, beta-site amyloid precursor protein (APP)-cleaving enzyme; APP, amyloid precursor protein; MIP-1α, macrophage inflammatory protein-1α; SD, Sprague–Dawley; PM_1_, PM with a diameter less than 1 μm; nanoPM, nanosized PM; WT, wild-type; EVs, extracellular vehicles; Nrf2, nuclear factor erythroid 2-related factor 2; PGE2, prosglandin E2; 4-HNE, 4-hydroxy-2-nonenal; 3-NT, 3-nitrotyrosine; PRRs, pattern recognition receptors; MAC1, macrophage antigen complex 1; CGNs, cerebellar granule neuron; DA, dopaminergic.

## References

[1] Costa L.G., Cole T.B., Dao K., Chang Y.C., Coburn J., Garrick J.M. (2020). Effects of air pollution on the nervous system and its possible role in neurodevelopmental and neurodegenerative disorders. Pharmacol. Ther..

[2] Ghio A.J., Smith C.B., Madden M.C. (2012). Diesel exhaust particles and airway inflammation. Curr. Opin. Pulm. Med..

[3] Wu W., Jin Y., Carlsten C. (2018). Inflammatory health effects of indoor and outdoor particulate matter. J. Allergy Clin. Immunol..

[4] Bell M.L., Dominici F., Ebisu K., Zeger S.L., Samet J.M. (2007). Spatial and temporal variation in PM_2.5_ chemical composition in the United States for health effects studies. Environ. Health Perspect..

[5] Gao J., Wang K., Wang Y., Liu S., Zhu C., Hao J., Liu H., Hua S., Tian H. (2018). Temporal-spatial characteristics and source apportionment of PM_2.5_ as well as its associated chemical species in the Beijing-Tianjin-Hebei region of China. Environ. Pollut..

[6] Shou Y., Huang Y., Zhu X., Liu C., Hu Y., Wang H. (2019). A review of the possible associations between ambient PM_2.5_ exposures and the development of Alzheimer’s disease. Ecotoxicol. Environ. Saf..

[7] Figueres C., Landrigan P.J., Fuller R. (2018). Tackling air pollution, climate change, and NCDs: Time to pull together. Lancet.

[8] Calderón-Garcidueñas L., Maronpot R.R., Torres-Jardon R., Henríquez-Roldán C., Schoonhoven R., Acuña-Ayala H., Villarreal-Calderón A., Nakamura J., Fernando R., Reed W. (2003). DNA damage in nasal and brain tissues of canines exposed to air pollutants is associated with evidence of chronic brain inflammation and neurodegeneration. Toxicol. Pathol..

[9] Calderón-Garcidueñas L., Reed W., Maronpot R.R., Henríquez-Roldán C., Delgado-Chavez R., Calderón-Garcidueñas A., Dragustinovis I., Franco-Lira M., Aragón-Flores M., Solt A.C. (2004). Brain inflammation and Alzheimer’s-like pathology in individuals exposed to severe air pollution. Toxicol. Pathol..

[10] Calderón-Garcidueñas L., Mora-Tiscareño A., Ontiveros E., Gómez-Garza G., Barragán-Mejía G., Broadway J., Chapman S., Valencia-Salazar G., Jewells V., Maronpot R.R. (2008). Air pollution, cognitive deficits and brain abnormalities: A pilot study with children and dogs. Brain Cogn..

[11] Fu P., Guo X., Cheung F.M.H., Yung K.K.L. (2019). The association between PM_2.5_ exposure and neurological disorders: A systematic review and meta-analysis. Sci. Total Environ..

[12] Wang J., Ma T., Ma D., Li H., Hua L., He Q., Deng X. (2021). The impact of air pollution on neurodegenerative diseases. Ther. Drug Monit..

[13] Lee K.H., Cha M., Lee B.H. (2020). Neuroprotective effect of antioxidants in the brain. Int. J. Mol. Sci..

[14] Juan C.A., Pérez de la Lastra J.M., Plou F.J., Pérez-Lebeña E. (2021). The chemistry of reactive oxygen species (ROS) revisited: Outlining their role in biological macromolecules (DNA, lipids and proteins) and induced pathologies. Int. J. Mol. Sci..

[15] Simpson D.S.A., Oliver P.L. (2020). ROS generation in microglia: Understanding oxidative stress and inflammation in neurodegenerative disease. Antioxidants.

[16] Block M.L., Zecca L., Hong J.S. (2007). Microglia-mediated neurotoxicity: Uncovering the molecular mechanisms. Nat. Rev. Neurosci..

[17] Block M.L., Calderón-Garcidueñas L. (2009). Air pollution: Mechanisms of neuroinflammation and CNS disease. Trends Neurosci..

[18] Genc S., Zadeoglulari Z., Fuss S.H., Genc K. (2012). The adverse effects of air pollution on the nervous system. J. Toxicol..

[19] Zhu X., Ji X., Shou Y., Huang Y., Hu Y., Wang H. (2020). Recent advances in understanding the mechanisms of PM_2.5_-mediated neurodegenerative diseases. Toxicol. Lett..

[20] Calderón-Garcidueñas L., Leray E., Heydarpour P., Torres-Jardón R., Reis J. (2016). Air pollution, a rising environmental risk factor for cognition, neuroinflammation and neurodegeneration: The clinical impact on children and beyond. Rev. Neurol..

[21] Greter M., Merad M. (2013). Regulation of microglia development and homeostasis. Glia.

[22] Wieghofer P., Knobeloch K.P., Prinz M. (2015). Genetic targeting of microglia. Glia.

[23] Priller J., Prinz M. (2019). Targeting microglia in brain disorders. Science.

[24] Mittelbronn M., Dietz K., Schluesener H.J., Meyermann R. (2001). Local distribution of microglia in the normal adult human central nervous system differs by up to one order of magnitude. Acta Neuropathol..

[25] Ong W.Y., Leong S.K., Garey L.J., Tan K.K., Zhang H.F. (1995). A light and electron microscopic study of HLA-DR positive cells in the human cerebral cortex and subcortical white matter. J. Hirnforsch..

[26] Lawson L.J., Perry V.H., Dri P., Gordon S. (1990). Heterogeneity in the distribution and morphology of microglia in the normal adult mouse brain. Neuroscience.

[27] Ueno M., Fujita Y., Tanaka T., Nakamura Y., Kikuta J., Ishii M., Yamashita T. (2013). Layer V cortical neurons require microglial support for survival during postnatal development. Nat. Neurosci..

[28] Jurga A.M., Paleczna M., Kuter K.Z. (2020). Overview of general and discriminating markers of differential microglia phenotypes. Front. Cell Neurosci..

[29] Zhang L., Zhang J., You Z. (2018). Switching of the Microglial Activation Phenotype Is a Possible Treatment for Depression Disorder. Front. Cell Neurosci..

[30] Cherry J.D., Olschowka J.A., O’Banion M.K. (2014). Neuroinflammation and M2 microglia: The good, the bad, and the inflamed. J. Neuroinflammation.

[31] Colonna M., Butovsky O. (2017). Microglia function in the central nervous system during health and neurodegeneration. Annu. Rev. Immunol..

[32] Subhramanyam C.S., Wang C., Hu Q., Dheen S.T. (2019). Microglia-mediated neuroinflammation in neurodegenerative diseases. Semin. Cell Dev. Biol..

[33] Du L., Zhang Y., Chen Y., Zhu J., Yang Y., Zhang H.L. (2017). Role of microglia in neurological disorders and their potentials as a therapeutic target. Mol. Neurobiol..

[34] Lee K.H., Cha M., Lee B.H. (2021). Crosstalk between neuron and glial cells in oxidative injury and neuroprotection. Int. J. Mol. Sci..

[35] Hanisch U.K., Kettenmann H. (2007). Microglia: Active sensor and versatile effector cells in the normal and pathologic brain. Nat. Neurosci..

[36] van Horssen J., Witte M.E., Schreibelt G., de Vries H.E. (2011). Radical changes in multiple sclerosis pathogenesis. Biochim. Biophys. Acta.

[37] Garza-Lombó C., Posadas Y., Quintanar L., Gonsebatt M.E., Franco R. (2018). Neurotoxicity linked to dysfunctional metal ion homeostasis and xenobiotic metal exposure: Redox signaling and oxidative stress. Antioxid. Redox Signal..

[38] Saijo K., Crotti A., Glass C.K. (2013). Regulation of microglia activation and deactivation by nuclear receptors. Glia.

[39] He Y., Gao Y., Zhang Q., Zhou G., Cao F., Yao S. (2020). IL-4 switches microglia/macrophage M1/M2 polarization and alleviates neurological damage by modulating the JAK1/STAT6 pathway following ICH. Neuroscience.

[40] Kwon H.S., Koh S.H. (2020). Neuroinflammation in neurodegenerative disorders: The roles of microglia and astrocytes. Transl. Neurodegener..

[41] Calderón-Garcidueñas L., Azzarelli B., Acuna H., Garcia R., Gambling T.M., Osnaya N., Monroy S., Delt M.R., Carson J.L., Villarreal-Calderon A. (2002). Air pollution and brain damage. Toxicol. Pathol..

[42] Calderón-Garcidueñas L., Solt A.C., Henríquez-Roldán C., Torres-Jardón R., Nuse B., Herritt L., Villarreal-Calderón R., Osnaya N., Stone I., García R. (2008). Long-term air pollution exposure is associated with neuroinflammation, an altered innate immune response, disruption of the blood-brain barrier, ultrafine particulate deposition, and accumulation of amyloid beta-42 and alpha-synuclein in children and young adults. Toxicol. Pathol..

[43] Calderón-Garcidueñas L., D’Angiulli A., Kulesza R.J., Torres-Jardón R., Osnaya N., Romero L., Keefe S., Herritt L., Brooks D.M., Avila-Ramirez J. (2011). Air pollution is associated with brainstem auditory nuclei pathology and delayed brainstem auditory evoked potentials. Int. J. Dev. Neurosci..

[44] Calderón-Garcidueñas L., Franco-Lira M., Henríquez-Roldán C., Osnaya N., González-Maciel A., Reynoso-Robles R., Villarreal-Calderon R., Herritt L., Brooks D., Keefe S. (2010). Urban air pollution: Influences on olfactory function and pathology in exposed children and young adults. Exp. Toxicol. Pathol..

[45] Riedel B.C., Thompson P.M., Brinton R.D. (2016). Age, APOE and sex: Triad of risk of Alzheimer’s disease. J. Steroid. Biochem. Mol. Biol..

[46] Calderón-Garcidueñas L., Kavanaugh M., Block M., D’Angiulli A., Delgado-Chávez R., Torres-Jardón R., González-Maciel A., Reynoso-Robles R., Osnaya N., Villarreal-Calderon R. (2012). Neuroinflammation, hyperphosphorylated tau, diffuse amyloid plaques, and down-regulation of the cellular prion protein in air pollution exposed children and young adults. J. Alzheimers Dis..

[47] Calderón-Garcidueñas L., González-Maciel A., Reynoso-Robles R., Kulesza R.J., Mukherjee P.S., Torres-Jardón R., Rönkkö T., Doty R.L. (2018). Alzheimer’s disease and alpha-synuclein pathology in the olfactory bulbs of infants, children, teens and adults ≤ 40 years in Metropolitan Mexico City. APOE4 carriers at higher risk of suicide accelerate their olfactory bulb pathology. Environ. Res..

[48] Cipriani G., Danti S., Carlesi C., Borin G. (2018). Danger in the air: Air pollution and cognitive dysfunction. Am. J. Alzheimers Dis Other Demen.

[49] Paul K.C., Haan M., Mayeda E.R., Ritz B.R. (2019). Ambient air pollution, noise, and late-life cognitive decline and dementia risk. Annu. Rev. Public Health.

[50] Power M.C., Adar S.D., Yanosky J.D., Weuve J. (2016). Exposure to air pollution as a potential contributor to cognitive function, cognitive decline, brain imaging, and dementia: A systematic review of epidemiologic research. Neurotoxicology.

[51] Costa L.G. (2017). Traffic-related air pollution and neurodegenerative diseases: Epidemiological and experimental evidence and potential underlying mechanisms-ScienceDirect. Adv. Neurotoxicol..

[52] Jung C.R., Lin Y.T., Hwang B.F. (2015). Ozone, particulate matter, and newly diagnosed Alzheimer’s disease: A population-based cohort study in Taiwan. J. Alzheimers Dis..

[53] Schikowski T., Vossoughi M., Vierkötter A., Schulte T., Teichert T., Sugiri D., Fehsel K., Tzivian L., Bae I.S., Ranft U. (2015). Association of air pollution with cognitive functions and its modification by APOE gene variants in elderly women. Environ. Res..

[54] Chen H., Kwong J.C., Copes R., Tu K., Villeneuve P.J., van Donkelaar A., Hystad P., Martin R.V., Murray B.J., Jessiman B. (2017). Living near major roads and the incidence of dementia, Parkinson’s disease, and multiple sclerosis: A population-based cohort study. Lancet.

[55] Zanobetti A., Dominici F., Wang Y., Schwartz J.D. (2014). A national case-crossover analysis of the short-term effect of PM_2.5_ on hospitalizations and mortality in subjects with diabetes and neurological disorders. Environ. Health.

[56] Kioumourtzoglou M.A., Schwartz J.D., Weisskopf M.G., Melly S.J., Wang Y., Dominici F., Zanobetti A. (2016). Long-term PM_2.5_ exposure and neurological hospital admissions in the Northeastern United States. Environ. Health Perspect..

[57] Calderón-Garcidueñas L., Avila-Ramírez J., Calderón-Garcidueñas A., González-Heredia T., Acuña-Ayala H., Chao C.K., Thompson C., Ruiz-Ramos R., Cortés-González V., Martínez-Martínez L. (2016). Cerebrospinal fluid biomarkers in highly exposed PM_2.5_ urbanites: The risk of Alzheimer’s and Parkinson’s diseases in young Mexico City residents. J. Alzheimers Dis..

[58] Blennow K., Dubois B., Fagan A.M., Lewczuk P., de Leon M.J., Hampel H. (2015). Clinical utility of cerebrospinal fluid biomarkers in the diagnosis of early Alzheimer’s disease. Alzheimers Dement..

[59] Calderón-Garcidueñas L., Mukherjee P.S., Waniek K., Holzer M., Chao C.K., Thompson C., Ruiz-Ramos R., Calderón-Garcidueñas A., Franco-Lira M., Reynoso-Robles R. (2018). Non-phosphorylated Tau in cerebrospinal fluid is a marker of Alzheimer’s disease continuum in young urbanites exposed to air pollution. J. Alzheimers Dis..

[60] Hou Y., Dan X., Babbar M., Wei Y., Hasselbalch S.G., Croteau D.L., Bohr V.A. (2019). Ageing as a risk factor for neurodegenerative disease. Nat. Rev. Neurol..

[61] Chen J.C., Wang X., Wellenius G.A., Serre M.L., Driscoll I., Casanova R., McArdle J.J., Manson J.E., Chui H.C., Espeland M.A. (2015). Ambient air pollution and neurotoxicity on brain structure: Evidence from women’s health initiative memory study. Ann. Neurol..

[62] Younan D., Petkus A.J., Widaman K.F., Wang X., Casanova R., Espeland M.A., Gatz M., Henderson V.W., Manson J.E., Rapp S.R. (2020). Particulate matter and episodic memory decline mediated by early neuroanatomic biomarkers of Alzheimer’s disease. Brain.

[63] Kirrane E.F., Bowman C., Davis J.A., Hoppin J.A., Blair A., Chen H., Patel M.M., Sandler D.P., Tanner C.M., Vinikoor-Imler L. (2015). Associations of Ozone and PM_2.5_ concentrations with Parkinson’s disease among participants in the Agricultural Health Study. J. Occup. Environ. Med..

[64] Liu R., Young M.T., Chen J.C., Kaufman J.D., Chen H. (2016). Ambient air pollution exposures and risk of Parkinson disease. Environ. Health Perspect..

[65] Palacios N., Fitzgerald K.C., Hart J.E., Weisskopf M.G., Schwarzschild M.A., Ascherio A., Laden F. (2014). Particulate matter and risk of Parkinson disease in a large prospective study of women. Environ. Health.

[66] Calderón-Garcidueñas L., Calderón-Garcidueñas A., Torres-Jardón R., Avila-Ramírez J., Kulesza R.J., Angiulli A.D. (2015). Air pollution and your brain: What do you need to know right now. Prim. Health Care Res. Dev..

[67] Xu X., Ha S.U., Basnet R. (2016). A review of epidemiological research on adverse neurological effects of exposure to ambient air pollution. Front. Public Health.

[68] Delgado-Saborit J.M., Guercio V., Gowers A.M., Shaddick G., Fox N.C., Love S. (2021). A critical review of the epidemiological evidence of effects of air pollution on dementia, cognitive function and cognitive decline in adult population. Sci. Total Environ..

[69] Calderón-Garcidueñas L., Cross J.V., Franco-Lira M., Aragón-Flores M., Kavanaugh M., Torres-Jardón R., Chao C.K., Thompson C., Chang J., Zhu H. (2013). Brain immune interactions and air pollution: Macrophage inhibitory factor (MIF), prion cellular protein (PrP(C)), Interleukin-6 (IL-6), interleukin 1 receptor antagonist (IL-1Ra), and interleukin-2 (IL-2) in cerebrospinal fluid and MIF in serum differentiate urban children exposed to severe vs. low air pollution. Front. Neurosci..

[70] Cox G.M., Kithcart A.P., Pitt D., Guan Z., Alexander J., Williams J.L., Shawler T., Dagia N.M., Popovich P.G., Satoskar A.R. (2013). Macrophage migration inhibitory factor potentiates autoimmune-mediated neuroinflammation. J. Immunol..

[71] Tang S., Li T., Fang J., Chen R., Cha Y., Wang Y., Zhu M., Zhang Y., Chen Y., Du Y. (2021). The exposome in practice: An exploratory panel study of biomarkers of air pollutant exposure in Chinese people aged 60–69 years (China BAPE Study). Environ. Int..

[72] Song J., Qu R., Sun B., Chen R., Kan H., An Z., Jiang J., Li J., Zhang Y., Wu W. (2022). Associations of short-term exposure to fine particulate matter with neural damage biomarkers: A panel study of healthy retired adults. Environ. Sci. Technol..

[73] Kilian J., Kitazawa M. (2018). The emerging risk of exposure to air pollution on cognitive decline and Alzheimer’s disease-Evidence from epidemiological and animal studies. Biomed. J..

[74] Costa L.G., Cole T.B., Coburn J., Chang Y.C., Dao K., Roque P. (2014). Neurotoxicants are in the air: Convergence of human, animal, and in vitro studies on the effects of air pollution on the brain. Biomed. Res. Int..

[75] Akiyama H., Barger S., Barnum S., Bradt B., Bauer J., Cole G.M., Cooper N.R., Eikelenboom P., Emmerling M., Fiebich B.L. (2000). Inflammation and Alzheimer’s disease. Neurobiol. Aging.

[76] Calderón-Garcidueñas L., Reynoso-Robles R., Vargas-Martínez J., Gómez-Maqueo-Chew A., Pérez-Guillé B., Mukherjee P.S., Torres-Jardón R., Perry G., Gónzalez-Maciel A. (2016). Prefrontal white matter pathology in air pollution exposed Mexico City young urbanites and their potential impact on neurovascular unit dysfunction and the development of Alzheimer’s disease. Environ. Res..

[77] Bhatt D.P., Puig K.L., Gorr M.W., Wold L.E., Combs C.K. (2015). A pilot study to assess effects of long-term inhalation of airborne particulate matter on early Alzheimer-like changes in the mouse brain. PLoS ONE.

[78] Ku T., Li B., Gao R., Zhang Y., Yan W., Ji X., Li G., Sang N. (2017). NF-κB-regulated microRNA-574-5p underlies synaptic and cognitive impairment in response to atmospheric PM_2.5_ aspiration. Part. Fibre Toxicol..

[79] Bai K.J., Chuang K.J., Chen C.L., Jhan M.K., Hsiao T.C., Cheng T.J., Chang L.T., Chang T.Y., Chuang H.C. (2019). Microglial activation and inflammation caused by traffic-related particulate matter. Chem. Biol. Interact..

[80] Bos I., De Boever P., Emmerechts J., Buekers J., Vanoirbeek J., Meeusen R., Van Poppel M., Nemery B., Nawrot T., Panis L.I. (2012). Changed gene expression in brains of mice exposed to traffic in a highway tunnel. Inhal. Toxicol..

[81] Hesterberg T.W., Long C.M., Lapin C.A., Hamade A.K., Valberg P.A. (2010). Diesel exhaust particulate (DEP) and nanoparticle exposures: What do DEP human clinical studies tell us about potential human health hazards of nanoparticles?. Inhal. Toxicol..

[82] Gerlofs-Nijland M.E., van Berlo D., Cassee F.R., Schins R.P., Wang K., Campbell A. (2010). Effect of prolonged exposure to diesel engine exhaust on proinflammatory markers in different regions of the rat brain. Part. Fibre Toxicol..

[83] van Berlo D., Albrecht C., Knaapen A.M., Cassee F.R., Gerlofs-Nijland M.E., Kooter I.M., Palomero-Gallagher N., Bidmon H.J., van Schooten F.J., Krutmann J. (2010). Comparative evaluation of the effects of short-term inhalation exposure to diesel engine exhaust on rat lung and brain. Arch. Toxicol..

[84] Levesque S., Taetzsch T., Lull M.E., Kodavanti U., Stadler K., Wagner A., Johnson J.A., Duke L., Kodavanti P., Surace M.J. (2011). Diesel exhaust activates and primes microglia: Air pollution, neuroinflammation, and regulation of dopaminergic neurotoxicity. Environ. Health Perspect..

[85] Levesque S., Surace M.J., McDonald J., Block M.L. (2011). Air pollution and the brain: Subchronic diesel exhaust exposure causes neuroinflammation and elevates early markers of neurodegenerative disease. J. Neuroinflamm..

[86] Durga M., Devasena T., Rajasekar A. (2015). Determination of LC50 and sub-chronic neurotoxicity of diesel exhaust nanoparticles. Environ. Toxicol. Pharmacol..

[87] Cacciottolo M., Wang X., Driscoll I., Woodward N., Saffari A., Reyes J., Serre M.L., Vizuete W., Sioutas C., Morgan T.E. (2017). Particulate air pollutants, APOE alleles and their contributions to cognitive impairment in older women and to amyloidogenesis in experimental models. Transl. Psychiatry.

[88] Costa L.G., Cole T.B., Coburn J., Chang Y.C., Dao K., Roqué P.J. (2017). Neurotoxicity of traffic-related air pollution. Neurotoxicology.

[89] Coburn J.L., Cole T.B., Dao K.T., Costa L.G. (2018). Acute exposure to diesel exhaust impairs adult neurogenesis in mice: Prominence in males and protective effect of pioglitazone. Arch. Toxicol..

[90] Cacciottolo M., Morgan T.E., Saffari A.A., Shirmohammadi F., Forman H.J., Sioutas C., Finch C.E. (2020). Traffic-related air pollutants (TRAP-PM) promote neuronal amyloidogenesis through oxidative damage to lipid rafts. Free Radic. Biol. Med..

[91] Cheng H., Saffari A., Sioutas C., Forman H.J., Morgan T.E., Finch C.E. (2016). Nanoscale particulate matter from urban traffic rapidly induces oxidative stress and inflammation in olfactory epithelium with concomitant effects on brain. Environ. Health Perspect..

[92] Chen X., Liu S., Zhang W., Wu C., Liu H., Zhang F., Lu Z., Ding W. (2018). Nrf2 deficiency exacerbates PM_2.5_-induced olfactory bulb injury. Biochem. Biophys. Res. Commun..

[93] Chen X., Guo J., Huang Y., Liu S., Huang Y., Zhang Z., Zhang F., Lu Z., Li F., Zheng J.C. (2020). Urban airborne PM_2.5_-activated microglia mediate neurotoxicity through glutaminase-containing extracellular vesicles in olfactory bulb. Environ. Pollut..

[94] Chen R., Gu X., Wang X. (2022). α-Synuclein in Parkinson’s disease and advances in detection. Clin. Chim. Acta.

[95] Thiankhaw K., Chattipakorn N., Chattipakorn S.C. (2022). PM_2.5_ exposure in association with AD-related neuropathology and cognitive outcomes. Environ. Pollut..

[96] Sama P., Long T.C., Hester S., Tajuba J., Parker J., Chen L.C., Veronesi B. (2007). The cellular and genomic response of an immortalized microglia cell line (BV2) to concentrated ambient particulate matter. Inhal. Toxicol..

[97] Kim R.E., Shin C.Y., Han S.H., Kwon K.J. (2020). Astaxanthin suppresses PM_2.5_-induced neuroinflammation by regulating Akt phosphorylation in BV-2 microglial cells. Int. J. Mol. Sci..

[98] Wang B.R., Shi J.Q., Ge N.N., Ou Z., Tian Y.Y., Jiang T., Zhou J.S., Xu J., Zhang Y.D. (2018). PM_2.5_ exposure aggravates oligomeric amyloid beta-induced neuronal injury and promotes NLRP3 inflammasome activation in an in vitro model of Alzheimer’s disease. J. Neuroinflamm..

[99] Zhang P., Hatter A., Liu B. (2007). Manganese chloride stimulates rat microglia to release hydrogen peroxide. Toxicol. Lett..

[100] Block M.L., Wu X., Pei Z., Li G., Wang T., Qin L., Wilson B., Yang J., Hong J.S., Veronesi B. (2004). Nanometer size diesel exhaust particles are selectively toxic to dopaminergic neurons: The role of microglia, phagocytosis, and NADPH oxidase. FASEB J..

[101] Roqué P.J., Dao K., Costa L.G. (2016). Microglia mediate diesel exhaust particle-induced cerebellar neuronal toxicity through neuroinflammatory mechanisms. Neurotoxicology.

[102] Cardona A.E., Pioro E.P., Sasse M.E., Kostenko V., Cardona S.M., Dijkstra I.M., Huang D., Kidd G., Dombrowski S., Dutta R. (2006). Control of microglial neurotoxicity by the fractalkine receptor. Nat. Neurosci..

[103] Ajmani G.S., Suh H.H., Pinto J.M. (2016). Effects of Ambient Air Pollution Exposure on Olfaction: A Review. Environ. Health Perspect..

[104] Lin M.T., Beal M.F. (2006). Mitochondrial dysfunction and oxidative stress in neurodegenerative diseases. Nature.

[105] Mutlu E.A., Engen P.A., Soberanes S., Urich D., Forsyth C.B., Nigdelioglu R., Chiarella S.E., Radigan K.A., Gonzalez A., Jakate S. (2011). Particulate matter air pollution causes oxidant-mediated increase in gut permeability in mice. Part Fibre Toxicol..

[106] Hosang L., Canals R.C., van der Flier F.J., Hollensteiner J., Daniel R., Flügel A., Odoardi F. (2022). The lung microbiome regulates brain autoimmunity. Nature.

[107] Jack C.S., Arbour N., Manusow J., Montgrain V., Blain M., McCrea E., Shapiro A., Antel J.P. (2005). TLR signaling tailors innate immune responses in human microglia and astrocytes. J. Immunol..

[108] Olson J.K., Miller S.D. (2004). Microglia initiate central nervous system innate and adaptive immune responses through multiple TLRs. J. Immunol..

[109] Husemann J., Loike J.D., Kodama T., Silverstein S.C. (2001). Scavenger receptor class B type I (SR-BI) mediates adhesion of neonatal murine microglia to fibrillar beta-amyloid. J. Neuroimmunol..

[110] Qin L., Liu Y., Wang T., Wei S.J., Block M.L., Wilson B., Liu B., Hong J.S. (2004). NADPH oxidase mediates lipopolysaccharide-induced neurotoxicity and proinflammatory gene expression in activated microglia. J. Biol. Chem..

[111] Pinti M., Ferraro D., Nasi M. (2021). Microglia activation: A role for mitochondrial DNA?. Neural Regen. Res..

[112] Agrawal I., Jha S. (2020). Mitochondrial Dysfunction and Alzheimer’s Disease: Role of Microglia. Front. Aging Neurosci..

[113] Pósfai B., Cserép C., Orsolits B., Dénes Á. (2019). New insights into microglia-neuron interactions: A neuron’s perspective. Neuroscience.

[114] Kim Y.S., Choi J., Yoon B.E. (2020). Neuron-Glia Interactions in Neurodevelopmental Disorders. Cells.

